# Diagnostic value and prognostic evaluation of Presepsin for sepsis in an emergency department

**DOI:** 10.1186/cc13070

**Published:** 2013-10-20

**Authors:** Bo Liu, Yun-Xia Chen, Qin Yin, Yun-Zhou Zhao, Chun-Sheng Li

**Affiliations:** 1 Emergency Department, Beijing Chao-yang Hospital, Capital Medical University, 8# Worker’s Stadium South Road, Beijing, Chao-yang District 100020, China

## Abstract

**Introduction:**

Presepsin levels are known to be increased in sepsis. The aim of this study was to evaluate the early diagnostic and prognostic value of Presepsin compared with procalcitonin (PCT), Mortality in Emergency Department Sepsis (MEDS) score and Acute Physiology and Chronic Health Evaluation II (APACHE II) score in septic patients in an emergency department (ED) and to investigate Presepsin as a new biomarker of sepsis.

**Methods:**

This study enrolled 859 consecutive patients with at least two diagnostic criteria for systemic inflammatory response syndrome (SIRS) who were admitted to Beijing Chao-yang Hospital ED from December 2011 to October 2012, and 100 age-matched healthy controls. Patients were stratified into four groups: SIRS, sepsis, severe sepsis, and septic shock. Plasma Presepsin and serum PCT were measured, and MEDS score and APACHE II score were calculated at enrollment. Comparisons were analyzed using the Kruskal-Wallis and Mann–Whitney *U* tests.

**Results:**

On admission, the median levels of plasma Presepsin increased with sepsis severity. The areas under the receiver operating characteristic (AUC) curves of Presepsin were greater than those of PCT in diagnosing sepsis, and predicting severe sepsis and septic shock. The AUC of Presepsin for predicting 28-day mortality in septic patients was slightly lower than that of PCT, MEDS score and APACHE II score. The AUC of a combination of Presepsin and MEDS score or APACHE II score was significantly higher than that of MEDS score or APACHE II score alone in predicting severe sepsis, and was markedly higher than that of Presepsin alone in predicting septic shock and 28-day mortality in septic patients, respectively. Plasma Presepsin levels in septic patients were significantly higher in non-survivors than in survivors at 28 days’ follow-up. Presepsin, MEDS score and APACHE II score were found to be independent predictors of severe sepsis, septic shock and 28-day mortality in septic patients. The levels of plasma Presepsin were positively correlated with PCT, MEDS score and APACHE II score in every septic group.

**Conclusion:**

Presepsin is a valuable biomarker for early diagnosis of sepsis, risk stratification, and evaluation of prognosis in septic patients in the ED.

## Introduction

Sepsis still represents a major cause of morbidity and mortality in critically ill patients despite the use of modern antibiotics and resuscitation therapies [1]. There is a lack of early diagnosis and timely intervention for sepsis in the emergency department (ED), and recent interest has focused on biomarkers for early diagnosis, risk stratification, and evaluation of prognosis of sepsis.

CD14, the high-affinity receptor for lipopolysaccharide/lipopolysaccharide binding protein complexes, is a glycoprotein expressed in macrophage, monocyte, and granulocyte cells and their cell membranes [2]. The lipopolysaccharide–lipopolysaccharide binding protein–CD14 complex is released into circulation by shedding of CD14 from the cell membrane, yielding soluble CD14. Presepsin (soluble CD14-ST), a novel biomarker for diagnosing sepsis, is a subtype of soluble CD14, and is a 13 kDa protein that is a truncated N-terminal fragment of CD14 [3].

Although some clinical studies confirmed that plasma presepsin levels were significantly increased in septic patients, and were positively correlated with the severity of sepsis, the sample sizes were relatively small [4-9], and these findings have not yet been corroborated in a large number of septic patients in the ED. The aim of this study was to investigate the clinical value of presepsin in early diagnosis, risk stratification and prognostic evaluation of sepsis in a large sample of septic patients in a hospital ED, and to compare it with the prognostic value of procalcitonin (PCT), the Mortality in Emergency Department Sepsis (MEDS) score and the Acute Physiology and Chronic Health Evaluation (APACHE) II score.

## Methods

### Patient inclusion and exclusion criteria

This prospective study was conducted in the ED of Beijing Chao-yang Hospital, a university teaching hospital with approximately 240,000 to 260,000 ED admissions per year. From December 2011 to October 2012, consecutive patients who fulfilled the criteria for sepsis as defined by the American College of Chest Physicians/Society of Critical Care Medicine (ACCP/SCCM) were enrolled [10]. Patients were classified at the time of enrollment as having sterile systemic inflammatory response syndrome (SIRS), sepsis, severe sepsis and septic shock, according to ACCP/SCCM criteria [10].

Exclusion criteria were as follows: <18 years old, terminal stage of disease (malignant cancer of any type, acquired immunodeficiency syndrome, end-stage liver or renal disease), and the patient or relatives did not consent to inclusion. Finally, after excluding 92 patients who did not meet the inclusion criteria and 49 patients who were lost to follow-up, 859 patients were enrolled and were followed for 28 days or until death. At the same time, 100 age-matched healthy individuals were enrolled as controls. This study was approved by the Beijing Chao-yang Hospital Ethics Committee. Written informed consent was obtained from every subject.

### Establishment of infection

The infections of different diseases in our study were clinically established on the basis of clinical features, laboratory findings, and imaging tests according to criteria of the International Sepsis Forum Consensus Conference on Definitions of Infection [11]. For example, the diagnosis of community-acquired pneumonia was mainly based on a new infiltrate plus at least one recently acquired respiratory symptom (cough, sputum production, dyspnea, tachypnea, pleuritic pain) or sign (auscultatory findings of abnormal breath sounds and rales). Intra-abdominal infections comprised the following diseases in our study: the diagnosis of peritonitis was based on clinical findings including abdominal pain, tenderness to palpation, and peritoneal signs such as rigidity or rebound tenderness, which were supported by radiographic findings, such as free air under the diaphragm or localized fluid collection visualized by computed tomography with a compatible clinical illness; the diagnosis of biliary tract infection was on the basis of clinical evidence of biliary tract infection with surgical or radiographic evidence of supportive complications; typhlitis was diagnosed according to a compatible clinical presentation with radiographic evidence of bowel wall edema and/or gas and/or hemorrhagic necrosis within the bowel wall of the cecum; and the diagnosis of pyelonephritis was based on clinical features (fever (>38°C), urgency, localized pain or tenderness at involved site), and pyuria, hematuria, and radiographic evidence of infection. Skin and soft-tissue infections (cellulitis) were suggested by the presence of a rapidly expanding erythema, local tenderness, pain, swelling, lymphangitis, and lymphadenopathy, which is frequently accompanied by systemic signs and symptoms including malaise, fever (temperature >38.0°C), and chills. Determination of bacterial meningitis was based on compatible clinical features, and cerebrospinal fluid findings (cerebrospinal fluid leukocyte count >1,000/mm^3^) [12].

### Data collection

Subject data including name, age, sex, past medical history and vital signs were recorded at enrollment. Laboratory examinations, including whole blood leukocyte counts, blood gas analysis, blood biochemistry, X-ray scans and others, were carried out within 24 hours.

Venous blood samples were obtained at ED admission and collected in tubes containing heparin or ethylenediamine tetraacetate and stored at –80°C after collection for analysis within 24 hours. Plasma presepsin concentrations were determined with a compact automated immunoanalyzer (PATHFAST; Mitsubishi Chemical Medience Corporation, Tokyo, Japan) based on a chemiluminescent enzyme immunoassay [5,6,13], and the assay results were obtained within 17 minutes. This assay has a normal reference range of 60 to 365 pg/ml.

PCT was measured by a BioMerieux Mini VIDAS immunoassay analyzer (Block Scientific, Bohemia, NY, USA) in serum samples. Serum was separated by centrifugation at 3,000 rpm for 5 minutes and analyzed within 24 hours. The upper and lower detection limits were 200.0 ng/ml and 0.05 ng/ml, respectively.

The MEDS score and APACHE II score were calculated according to age, past medical history, vital signs and laboratory results [14,15] when the patients were admitted to the ED.

Septic patients were classified into surviving and nonsurviving groups according to 28-day survival. The enrolled septic patients who died from all causes within the follow-up time were considered nonsurvivors.

### Statistical analysis

All data were analyzed by SPSS 16.0 software (SPSS Inc., Chicago, IL, USA). Plasma presepsin levels, serum PCT levels, MEDS score and APACHE II score had skewed distributions and were expressed as the median (25th to 75th percentile). For multi-group comparisons, Kruskal–Wallis one-way analysis of variance was applied, and two-group comparisons were performed nonparametrically using the Mann–Whitney U test. To compare the predictive value of presepsin, PCT, MEDS score and APACHE II score for severe sepsis, septic shock and 28-day mortality, receiver operating characteristic (ROC) curves were constructed and the areas under the ROC curves (AUCs) were determined. The outcome variable was 28-day mortality. On the basis of optimal thresholds determined according to ROC curve analysis, prognostic parameters (sensitivity, specificity, positive predictive value (PPV), negative predictive value (NPV), positive likelihood ratio (LR+) and negative likelihood ratio (LR–)) were also calculated. For comparisons of AUCs, the *Z*-test formula was applied:

$$Z=\left(A1‒A2\right)/\sqrt{{\mathrm{SE}}_{1}{}^{2}+{\mathrm{SE}}_{2}{}^{2}}$$

The test standard was *Z*_0.05_ = 1.96, *Z*_0.01_ = 2.58. Binary logistic regression analysis was applied to determine the independent predictors of severe sepsis, septic shock, and 28-day mortality. All statistical tests were two-tailed, and *P* <0.05 was considered statistically significant. Spearman correlation analysis was applied to determine the correlation between presepsin, PCT, MEDS score and APACHE II score.

## Results

### Characteristics of enrolled subjects

Between December 2011 and October 2012, 859 patients in the ED of Beijing Chao-yang Hospital and 100 healthy controls were enrolled in this study. No significant differences were found in age, sex and correlative diseases among the five groups (SIRS, sepsis, severe sepsis, septic shock, and control groups). The characteristics, diseases and associated infections in enrolled subjects are presented in Table 1.

**Table 1:** Patient characteristics

	**Control**	**SIRS**	**Sepsis**	**Severe sepsis**	**Septic shock**	***P *** **value**
*n*	100	179	372	210	98	
Age (years)	68	70	71	73	73	0.1
	(65 to 74)	(58 to 76)	(59 to 78)	(60 to 78)	(65 to 78)	
Male (%)	57.0	53.6	61.2	62.9	58.2	0.5
Presepsin (pg/ml)	130	212	325	787	1084	0.00
	(104 to 179)	(143 to 300)	(210 to 480)	(464 to 1249)	(695 to 2365)	
PCT (ng/ml)	0.05	0.05	0.17	1.09	6.99	0.00
	(0.05 to 0.07)	(0.05 to 0.23)	(0.05 to 0.79)	(0.21 to 7.52)	(0.88 to 38.26)	
MEDS score		6.5	8.0	14.0	19.0	0.00
		(3.0 to 10.0)	(7.0 to 10.0)	(10.0 to 16.0)	(16.8 to 22.0)	
APACHE II score		13.0	14.0	19.0	22.5	0.00
		(8.0 to 19.0)	(10.0 to 18.0)	(14.0 to 22.0)	(19.0 to 30.25)	
Diagnosis						
Respiratory		AECOPD (70)	Pneumonia (259)	Pneumonia (145)	Pneumonia (70)	0.92
		Asthma (38)				
		PE (12)				
Abdominal		Pancreatitis (34)	IAI (82)	IAI (52)	IAI (22)	0.42
Cerebral		Stroke (12)	Meningitis (12)	Meningitis (6)	Meningitis (4)	0.4
Urinary			Pyelonephritis (15)	Pyelonephritis (7)	Pyelonephritis (2)	0.9
Others		DKA (13)	Skin/soft tissue infection (4)			

Data are presented as median (25th to 75th percentile). AECOPD, acute exacerbation of chronic obstructive pulmonary diseases; APACHE, Acute Physiology and Chronic Health Evaluation; DKA, diabetic ketoacidosis; IAI, intraabdominal infection; MEDS, Mortality in Emergency Department Sepsis; PE, pulmonary embolism; PCT, procalcitonin; SIRS, systemic inflammatory response syndrome.

### Comparison of median levels of presepsin, procalcitonin, MEDS score and APACHE II score

**Figure 1:**
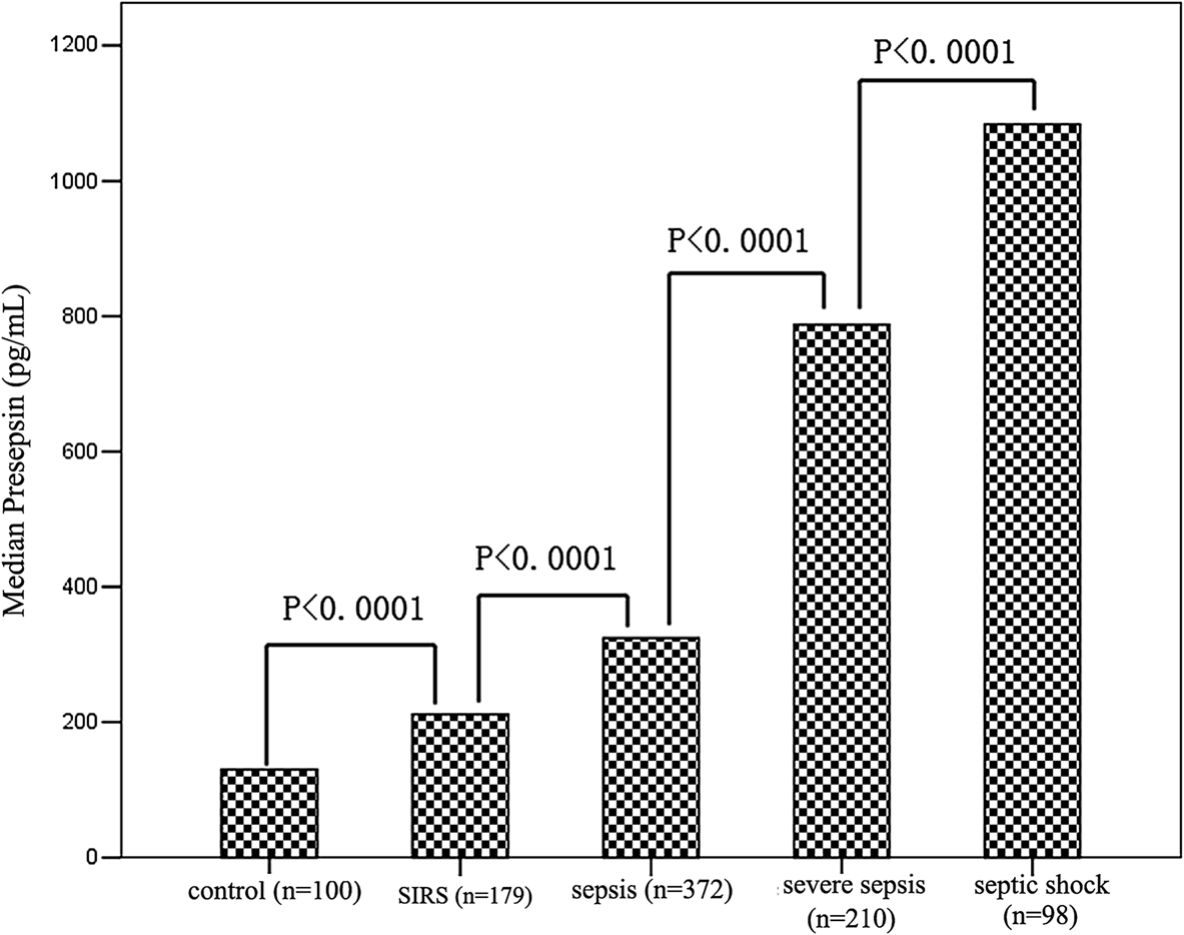
**Plasma presepsin levels in healthy control individuals, and patients with SIRS, sepsis, severe sepsis and septic shock at emergency department admission.** Columns represent median presepsin levels. Numbers of samples are indicated in parentheses. SIRS, systemic inflammatory response syndrome.

**Figure 2:**
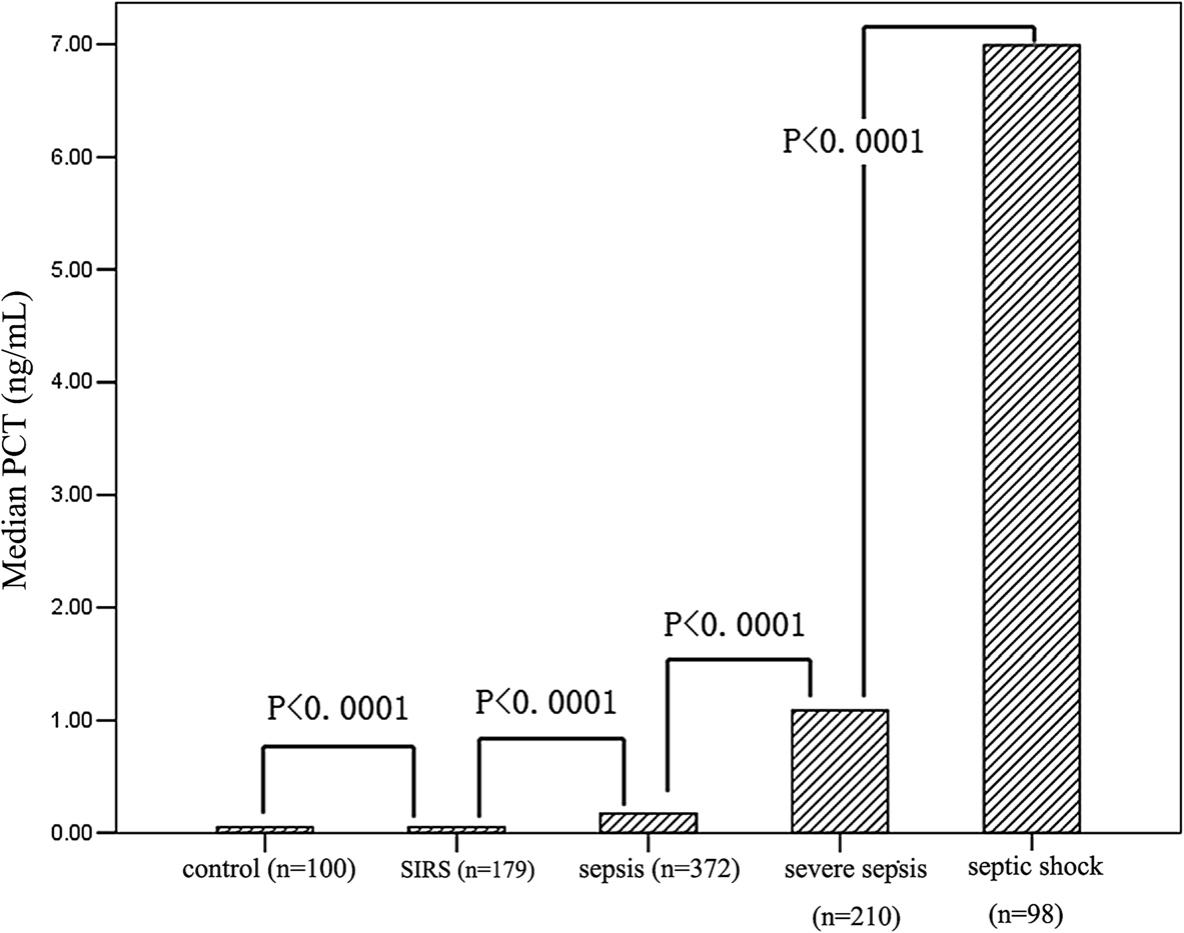
**Serum procalcitonin levels in healthy control individuals, and patients with SIRS, sepsis, severe sepsis and septic shock at emergency department admission.** Columns represent median procalcitonin (PCT) levels. Numbers of samples are indicated in parentheses. SIRS, systemic inflammatory response syndrome.

**Figure 3:**
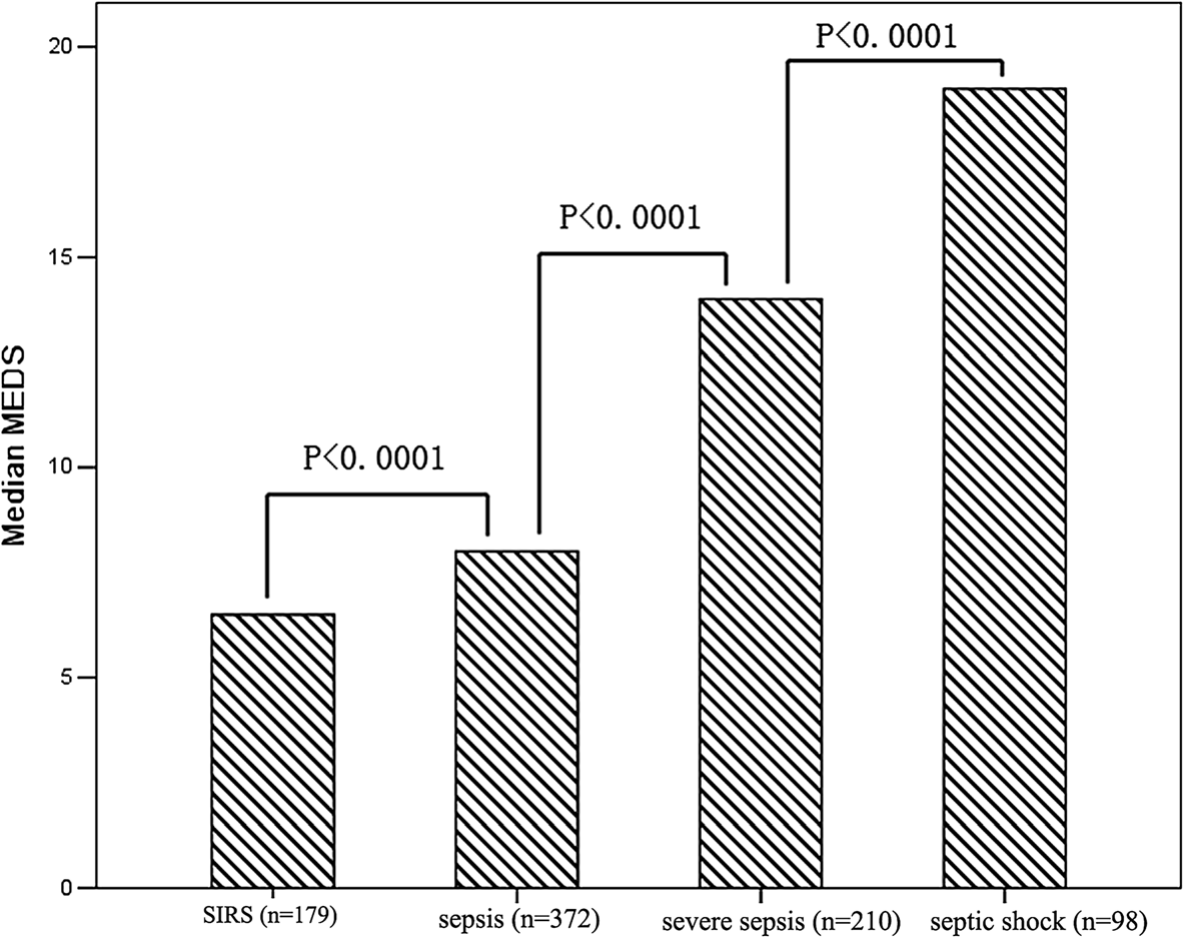
**MEDS score in patients with SIRS, sepsis, severe sepsis and septic shock at emergency department admission.** Columns represent median Mortality in Emergency Department Sepsis (MEDS) score levels. Numbers of samples are indicated in parentheses. SIRS, systemic inflammatory response syndrome.

**Figure 4:**
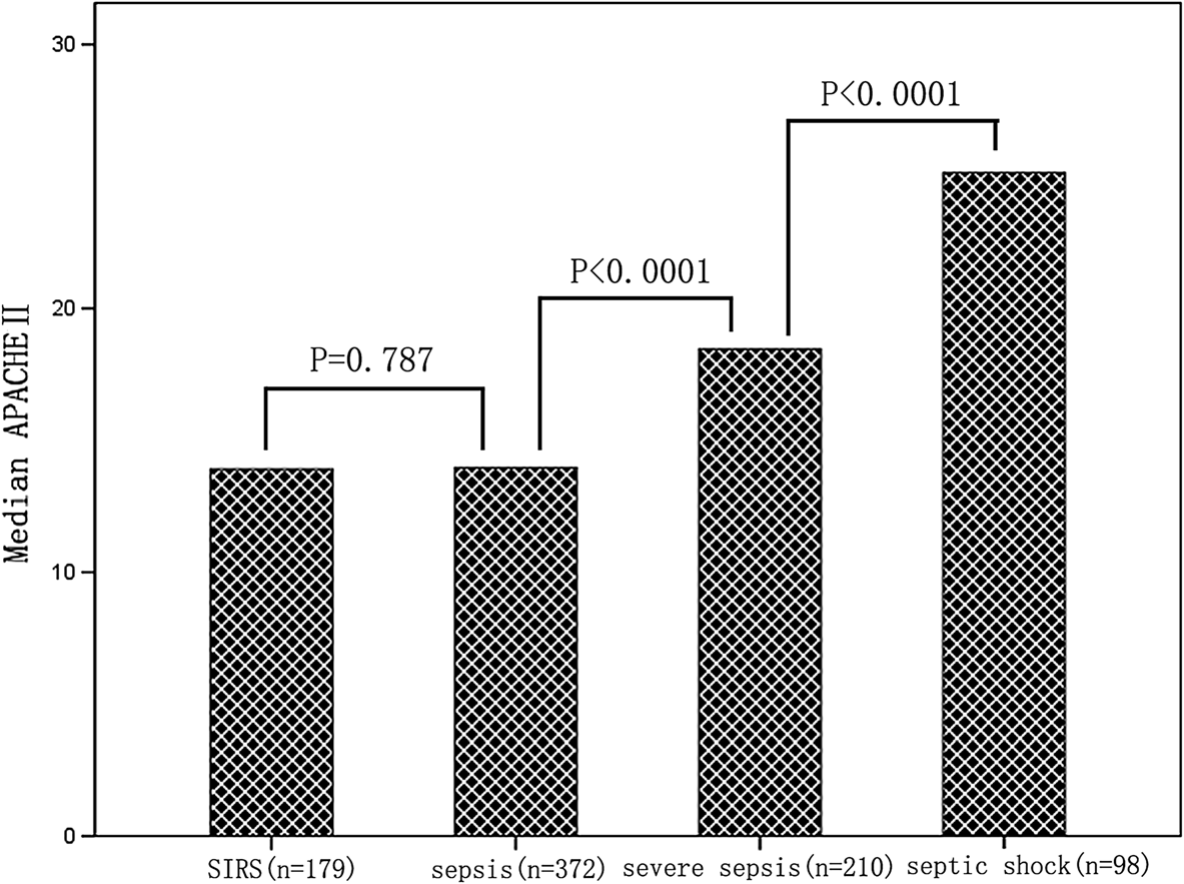
**APACHE II score in patients with SIRS, sepsis, severe sepsis and septic shock at emergency department admission.** Columns represent median Acute Physiology and Chronic Health Evaluation (APACHE) II score levels. Numbers of samples are indicated in parentheses. SIRS, systemic inflammatory response syndrome.

The median presepsin levels, PCT levels, MEDS score and APACHE II score in each group are shown in Table 1. Plasma presepsin, serum PCT, MEDS score and APACHE II score at ED admission were significantly different among these groups. Compared with the healthy control group, presepsin and PCT levels were significantly higher in patients with SIRS, sepsis, severe sepsis and septic shock (*P* <0.0001), and presepsin levels, PCT levels and MEDS score were significantly higher in sepsis, severe sepsis and septic shock than in SIRS (*P* <0.0001). The APACHE II score was significantly higher in severe sepsis and septic shock than in SIRS (*P* <0.0001), but there was no difference for APACHE II score in sepsis than in SIRS (*P* = 0.787). Presepsin levels, PCT levels, MEDS score and APACHE II score were markedly higher in severe sepsis and septic shock than in sepsis (*P* <0.0001), and were obviously higher in septic shock than in severe sepsis (*P* <0.0001). Median presepsin levels, PCT levels, MEDS score and APACHE II score among these groups are illustrated in Figures 1, 2, 3 and 4.

### Value of presepsin and procalcitonin for diagnosing sepsis

**Figure 5:**
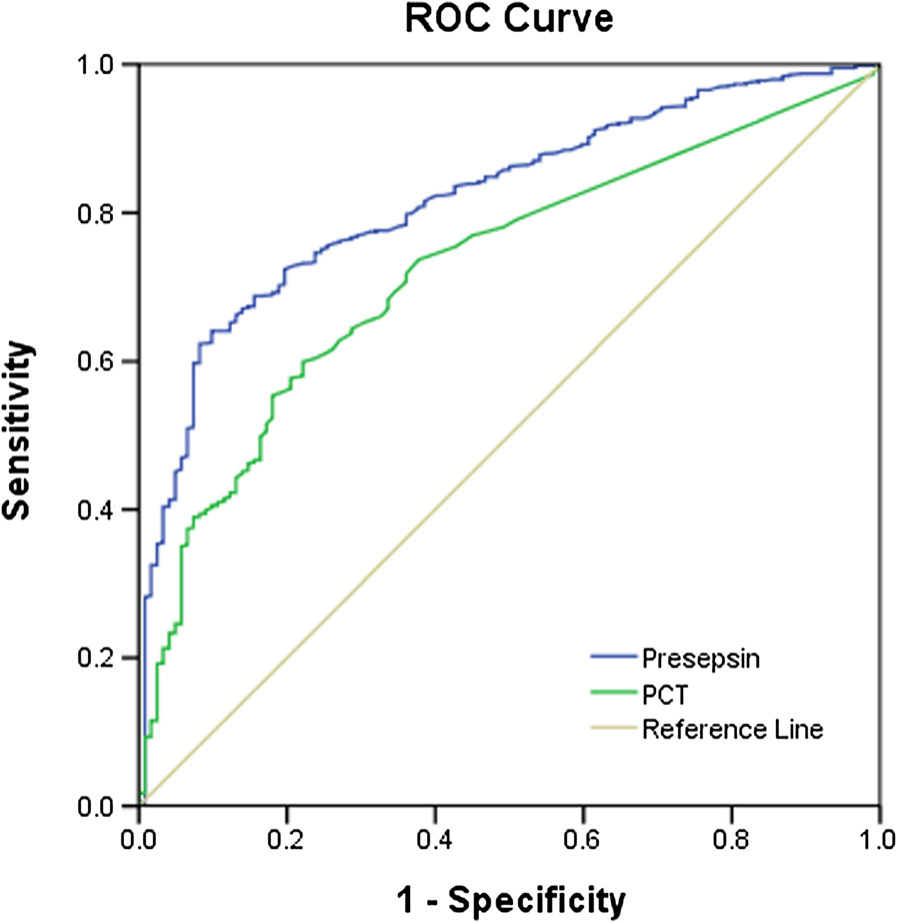
**Receiver operating characteristic curves of presepsin and procalcitonin for diagnosis of sepsis.** Areas under the receiver operating characteristic (ROC) curve: presepsin (blue line), 0.820 (95% confidence interval: 0.784 to 0.856), *P* < 0.0001; and procalcitonin (PCT; green line), 0.724 (95% confidence interval: 0.680 to 0.769), *P* <0.0001.

The ROC curves of presepsin and PCT for diagnosing sepsis among the five groups are shown in Figure 5. The AUC of presepsin was 0.820, significantly higher than that of PCT (0.724; *P* <0.01).

Using a presepsin cutoff value of 317 pg/ml for diagnosing sepsis, the sensitivity was 70.8%, the specificity was 85.8%, the PPV was 93.2%, the NPV was 51.6%, the predictive accuracy was 74.8%, the LR + was 4.99, and the LR– was 0.34.

Using a PCT cutoff value of 0.25 ng/ml for diagnosing sepsis, the sensitivity was 60.0%, the specificity was 77.7%, the PPV was 92.9%, the NPV was 28.4%, the predictive accuracy was 63.0%, the LR + was 2.69, and the LR– was 0.51.

### Value of presepsin, procalcitonin, MEDS score and APACHE II score for predicting severe sepsis

**Figure 6:**
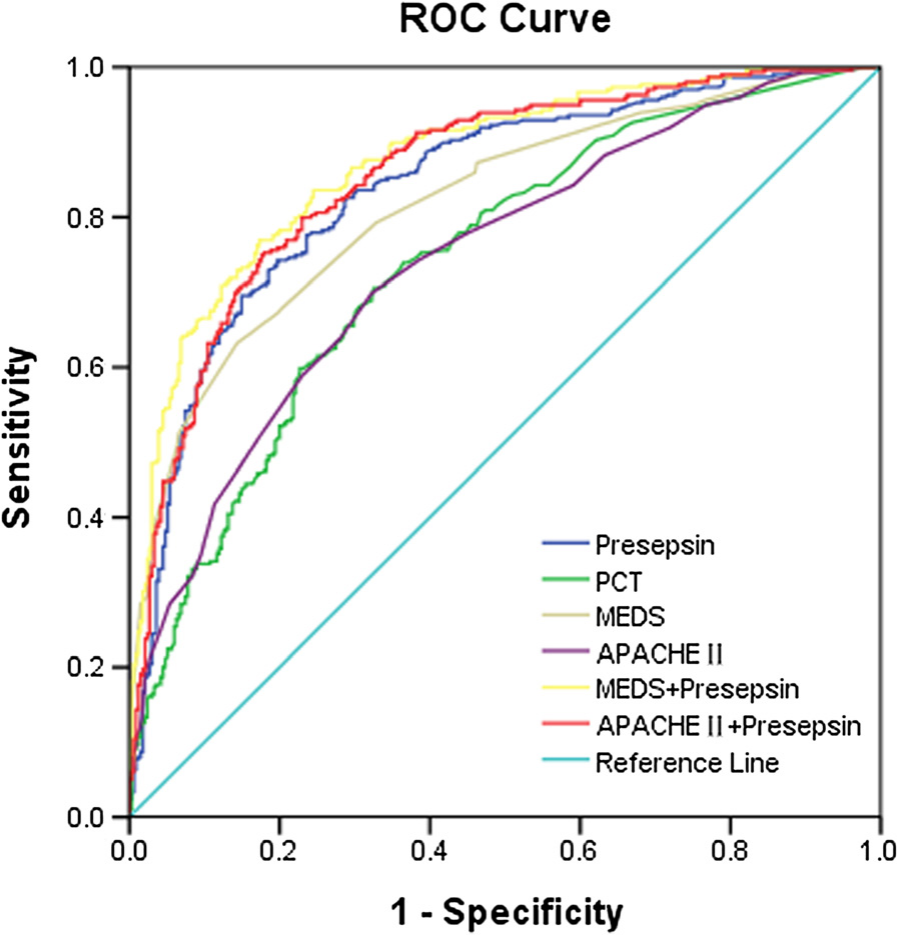
**Receiver operating characteristic curves of presepsin, procalcitonin, MEDS score and APACHE II score for predicting severe sepsis in septic patients.** Areas under the receiver operating characteristic (ROC) curves: presepsin (blue line), 0.840 (95% confidence interval (CI): 0.809 to 0.872), *P* <0.0001; procalcitonin (PCT; green line), 0.741 (95% CI: 0.703 to 0.779), *P* <0.0001; Mortality in Emergency Department Sepsis (MEDS) score (brown line), 0.818 (95% CI: 0.785 to 0.851), *P* <0.0001; Acute Physiology and Chronic Health Evaluation (APACHE) II score (purple line), 0.744 (95% CI: 0.706 to 0.782), *P* <0.0001; presepsin in combination with MEDS score (yellow line), 0.875 (95% CI: 0.848 to 0.901), *P* <0.0001; and presepsin in combination with APACHE II score (pink line), 0.858 (95% CI: 0.829 to 0.887), *P* <0.0001.

The ROC curves of presepsin, PCT, MEDS score and APACHE II score for predicting severe sepsis in septic patients are displayed in Figure 6. The AUC of presepsin was 0.840, significantly higher than that of PCT (0.741; *P* <0.01). The AUC of a combination of presepsin and MEDS score or APACHE II score was significantly higher than that of MEDS score or APACHE II score alone in predicting severe sepsis (0.875 vs. 0.818 or 0.858 vs. 0.744; all *P* <0.01), and there was no difference for the combination of presepsin and MEDS score or APACHE II score compared with presepsin alone (all *P* >0.05).

Using a presepsin cutoff value of 449 pg/ml for predicting severe sepsis, the sensitivity was 82.4%, the specificity was 72.4%, the PPV was 71.3%, the NPV was 83.2%, the predictive accuracy was 77.0%, the LR + was 2.99, and the LR– was 0.24. The detailed results are displayed in Tables 2 and 3.

**Table 2:** Areas under the curve of various parameters for predicting severe sepsis, septic shock and 28-day mortality in septic patients

	**Variable**	**AUC**	**Standard error**	***P *** **value**	**95% confidence interval**	
					**Lower limit**	**Upper limit**
Severe sepsis	Presepsin	0.840	0.016	0	0.809	0.872
	Procalcitonin	0.741	0.019	0	0.703	0.779
	MEDS score	0.818	0.017	0	0.785	0.851
	APACHE II score	0.744	0.019	0	0.706	0.782
	MEDS score + presepsin^a^	0.875	0.014	0	0.848	0.901
	APACHE II score + presepsin^b^	0.858	0.015	0	0.829	0.887
Septic shock	Presepsin	0.790	0.024	0	0.742	0.837
	Procalcitonin	0.768	0.024	0	0.721	0.816
	MEDS score	0.904	0.017	0	0.871	0.938
	APACHE II score	0.820	0.023	0	0.774	0.865
	MEDS score + presepsin^c^	0.924	0.014	0	0.897	0.951
	APACHE II score + presepsin^d^	0.868	0.019	0	0.831	0.905
28-day mortality	Presepsin	0.658	0.023	0	0.614	0.703
	Procalcitonin	0.679	0.022	0	0.636	0.722
	MEDS score	0.719	0.021	0	0.677	0.760
	APACHE II score	0.722	0.021	0	0.681	0.763
	MEDS score + presepsin^e^	0.731	0.021	0	0.690	0.771
	APACHE II score + presepsin^f^	0.734	0.021	0	0.693	0.775

AUC, area under the receiver operating characteristic curve. ^a^Compared with Mortality in Emergency Department Sepsis (MEDS) score, *P* <0.01. ^b^Compared with Acute Physiology and Chronic Health Evaluation (APACHE) II score, *P* <0.01. ^c^Compared with presepsin, *P* <0.01. ^d^Compared with presepsin, *P* < 0.05. ^e^Compared with presepsin, *P* <0.05. ^f^Compared with presepsin, *P* <0.05.

**Table 3:** Performance of multivariable models for predicting severe sepsis, septic shock and 28-day mortality in septic patients

	**Variable**	**Cutoff**	**Sensitivity (%)**	**Specificity (%)**	**PPV (%)**	**NPV (%)**	**Accuracy (%)**	**LR+**	**LR**–
Severe sepsis	Presepsin	449 pg/ml	82.4	72.4	71.3	83.2	77.0	2.99	0.24
	Procalcitonin	1.435 ng/ml	52.0	79.8	69.6	65.1	66.7	2.57	0.60
	MEDS score	13.5 score	63.0	86.3	79.2	73.8	75.8	4.60	0.42
	APACHE II score	16.5 score	69.8	68.5	64.8	73.3	69.1	2.22	0.44
Septic shock	Presepsin	550 pg/ml	85.7	63.6	28.5	96.3	66.8	2.35	0.22
	Procalcitonin	4.415 ng/ml	54.1	81.1	34.2	90.7	77.0	2.86	0.57
	MEDS score	16.5 score	75.5	90.0	56.1	95.6	87.9	7.55	0.27
	APACHE II score	20.5 score	70.4	78.4	35.4	94.0	77.2	3.26	0.38
28-day mortality	Presepsin	556 pg/ml	62.2	66.8	48.3	78.0	65.3	1.87	0.57
	Procalcitonin	1.125 ng/ml	54.2	70.0	47.7	75.1	64.7	1.81	0.65
	MEDS score	13.5 score	59.1	75.4	54.3	78.9	70.0	2.40	0.54
	APACHE II score	16.5 score	71.1	62.2	48.2	81.3	65.1	1.88	0.46

APACHE, Acute Physiology and Chronic Health Evaluation; LR+, positive likelihood ratio, LR–, negative likelihood ratio; MEDS, Mortality in Emergency Department Sepsis; NPV, negative predictive value; PPV, positive predictive value.

### Value of presepsin, procalcitonin, MEDS score and APACHE II score for predicting septic shock

The AUC of presepsin for predicting septic shock was 0.790, higher than that of PCT (0.768), but was not statistically significant (*P* >0.05). The AUC of a combination of presepsin and MEDS score or APACHE II score was significantly higher than that of presepsin alone in predicting septic shock (0.924 vs. 0.790, *P* < 0.01; 0.868 vs. 0.790, *P* < 0.05), and there was no difference for the combination of presepsin and MEDS score or APACHE II score compared with MEDS score or APACHE II score alone (all *P* >0.05), respectively. The AUC of a combination of presepsin and MEDS score was greater than that of a combination of presepsin and APACHE II score (*P* <0.05).

Using a presepsin cutoff value of 550 pg/ml for predicting septic shock, the sensitivity was 85.7%, the specificity was 63.6%, the PPV was 28.5%, the NPV was 96.3%, the predictive accuracy was 66.8%, the LR + was 2.35, and the LR– was 0.22. The detailed results are demonstrated in Tables 2 and 3.

### Value of presepsin, procalcitonin, MEDS score and APACHE II score for predicting 28-day mortality

The AUC of presepsin for predicting 28-day mortality in septic patients was 0.658, slightly lower than that of PCT (0.679; *P* >0.05), MEDS score (0.719; *P* >0.05), and APACHE II score (0.722; *P* <0.05). The AUC of presepsin in combination with MEDS score or APACHE II score was 0.731 or 0.734, which was more statistically significant compared with presepsin alone (0.658; all *P* <0.05), and there was no difference for the combination of presepsin and MEDS score or APACHE II score compared with MEDS score or APACHE II score alone (all *P* >0.05), respectively.

Using a presepsin cutoff value of 556 pg/ml for predicting 28-day mortality in septic patients, the sensitivity was 62.2%, the specificity was 66.8%, the PPV was 48.3%, the NPV was 78.0%, the predictive accuracy was 65.3%, the LR + was 1.87, and the LR– was 0.57. The detailed results are presented in Tables 2 and 3.

### Comparison of median levels of presepsin at admission in nonsurviving and surviving groups of septic patients at 28-day follow-up

**Figure 7:**
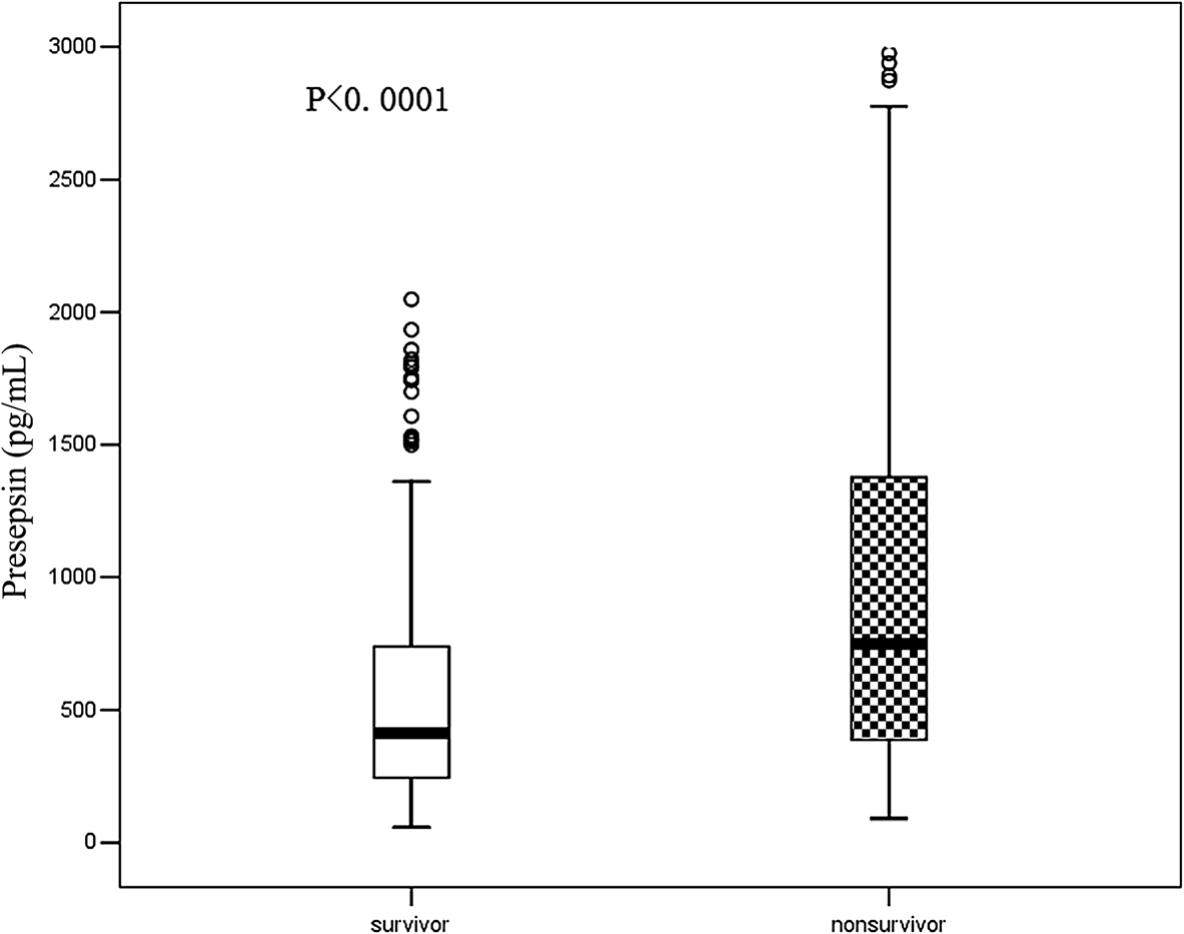
**Presepsin levels at admission in surviving and nonsurviving groups of septic patients at 28-day follow-up.** Lines denote median values, boxes represent 25th to 75th percentiles and whiskers indicate the range.

The median levels of presepsin were significantly higher in nonsurvivors than in survivors (748 pg/ml (385 to 1386 pg/ml) vs. 412 pg/ml (243 to 744 pg/ml); *P* <0.0001). The detailed results are illustrated in Figure 7.

### Presepsin, procalcitonin, MEDS score and APACHE II score as independent predictors of severe sepsis, septic shock and 28-day mortality

Using binary logistic regression analysis, presepsin (*B* = 0.001, odds ratio (OR) = 1.001, *P* = 0), MEDS score (*B* = 0.232, OR =1.262, *P* = 0) and APACHE II score (*B* = 0.081, OR =1.085, *P* = 0) were found to be the independent predictors of severe sepsis in septic patients, but PCT (*B* = 0.010, OR = 1.010, *P* = 0.069) was not (Table 4).

**Table 4:** Independent factors predicting severe sepsis, septic shock and 28-day mortality in septic patients

	**Variable**	***B***	**Standard error**	**Wald**	**Degrees of freedom**	***P *** **value**	**Odds ratio**	**95% confidence interval**	
								**Lower limit**	**Upper limit**
Severe sepsis	Presepsin	0.001	0.000	39.938	1	0.000	1.001	1.001	1.002
	Procalcitonin	0.010	0.005	3.298	1	0.069	1.010	0.999	1.021
	MEDS score	0.232	0.029	63.948	1	0.000	1.262	1.192	1.336
	APACHE II score	0.081	0.019	17.804	1	0.000	1.085	1.044	1.126
	Age	–0.028	0.009	9.607	1	0.002	0.973	0.956	0.990
	Gender	–1.101	0.224	0.204	1	0.651	0.904	0.582	1.403
	Constant	–3.223	0.697	21.374	1	0.000	0.040		
Septic shock	Presepsin	0.000	0.000	13.308	1	0.000	1.000	1.000	1.001
	Procalcitonin	0.008	0.004	5.014	1	0.025	1.008	1.001	1.025
	MEDS score	0.347	0.045	58.391	1	0.000	1.415	1.295	1.547
	APACHE II score	0.094	0.024	14.928	1	0.000	1.098	1.047	1.151
	Age	–0.027	0.012	5.285	1	0.022	0.973	0.951	0.996
	Gender	–0.081	0.325	0.062	1	0.803	0.922	0.487	1.744
	Constant	–7.462	1.083	47.511	1	0.000	0.001		
28-day mortality	Presepsin	0.000	0.000	5.296	1	0.021	1.000	1.000	1.000
	Procalcitonin	0.002	0.003	0.382	1	0.536	1.002	0.996	1.008
	MEDS score	0.088	0.022	15.223	1	0.000	1.091	1.045	1.141
	APACHE II score	0.080	0.016	25.658	1	0.000	1.083	1.050	1.117
	Age	0.004	0.007	0.346	1	0.556	1.004	0.990	1.019
	Gender	–0.038	0.194	0.038	1	0.846	0.963	0.658	1.409
	Constant	–3.651	0.623	34.307	1	0.000	0.026		

APACHE, Acute Physiology and Chronic Health Evaluation; MEDS, Mortality in Emergency Department Sepsis.

Presepsin (*B* = 0.000, OR = 1.000, *P* = 0), PCT (B = 0.008, OR = 1.008, *P* = 0.025), MEDS score (*B* = 0.347, OR = 1.415, *P* = 0) and APACHE II score (*B* = 0.094, OR =1.098, *P* = 0) were found to be the independent predictors of septic shock in septic patients (Table 4).

Presepsin (*B* = 0.000, OR = 1.000, *P* = 0.021), MEDS score (*B* = 0.088, OR = 1.091, *P* = 0) and APACHE II score (*B* = 0.080, OR =1.083, *P* = 0) were found to be independent predictors of 28-day mortality in septic patients, but PCT (*B* = 0.002, OR = 1.002, *P* = 0.536) was not (Table 4).

### Correlation of plasma presepsin levels with serum procalcitonin levels, MEDS score and APACHE II score in septic patients

Spearman correlation analysis of presepsin with PCT, MEDS score and APACHE II score showed that, compared with PCT, MEDS score and APACHE II score, the correlation coefficients of presepsin were 0.502, 0.414 and 0.294 (all *P* <0.0001), respectively, which suggested significantly positive correlations.

## Discussion

Despite the use of antimicrobial agents and advanced life support, the case fatality rate for patients with sepsis has remained between 20 and 30% during the past two decades [16,17].

Biomarkers play a pivotal pole in early diagnosis, differential diagnosis, risk stratification, therapy monitoring and evaluation of prognosis of sepsis. Presepsin, generated by circulating plasma proteases activating cleavage of soluble CD14 [18], is a novel biomarker that has been used for diagnosis of sepsis in recent years, and its production is associated with phagocytosis and cleavage of microorganisms by lysosomal enzymes [19].

In this study, we found that presepsin levels increased in early sepsis, and that the levels were significantly higher than in healthy controls and in SIRS patients, which was in accordance with previous results [4,5,9]. In addition, with progression of sepsis, the plasma presepsin levels increased accordingly, and reached the highest levels in septic shock. The present study demonstrated that plasma presepsin levels were a good parameter for reflecting the severity of sepsis.

More recently, PCT has also been used widely as a biomarker for sepsis diagnosis, risk stratification, evaluation of prognosis, and therapy monitoring. Most studies that have examined the discriminative ability of serum PCT in the diagnosis of sepsis have been small, with heterogeneity in the patients enrolled, and have used different cutoff values, and hence have provided variable results [20]. In this study, the cutoff value of PCT for diagnosing sepsis according to the ROC curve was 0.25 ng/ml, lower than that (0.5 ng/ml) commonly used in clinical settings [21,22]. Possible reasons for the difference were as follows: first, the SIRS and septic patients were enrolled according to ACCP/SCCM criteria, not PCT, which was only considered as a comparative index; second, the site of infection in the majority of enrolled patients was located in the lower respiratory tract, thus the PCT cutoff level of 0.25 ng/ml was usually used for diagnosing low respiratory tract infection [23,24]; third, the septic patients were enrolled and blood samples were obtained at the time of ED admission, when the concentration of PCT had not increased fully or reached a maximum; and fourth, the septic patients may have been treated with antimicrobial agents outside hospital, which may affect the results for PCT, as well as those for presepsin. Furthermore, the median levels of PCT in the present study were lower than the commonly used cutoff values in different clinical settings (Table 1), and the cutoff level of presepsin (317 pg/ml) was lower than the previously reported cutoff values of 400 to 600 pg/ml for diagnosing sepsis [5,9].

Although serum PCT can be used as a biomarker in the diagnosis of sepsis, PCT is also increased in other conditions, such as multiple trauma, extensive burns, pancreatitis, organ transplantation, major surgery, and SIRS, but not in infection [25], and its PPV and NPV values are not sufficient to rule in or rule out sepsis in a standalone test. A recent meta-analysis found that the diagnostic performance of PCT was low, with 71% (95% confidence interval 67 to 76%) sensitivity and specificity for serum PCT as a biomarker of sepsis. PCT therefore cannot reliably differentiate sepsis from other conditions in critically ill adult patients [26].

Compared with PCT, presepsin is a highly specific biomarker for diagnosing bacterial infections because it is produced in association with bacterial phagocytosis. Presepsin was shown to be secreted from granulocytes by infectious stimuli in an animal sepsis model [19]. In this study, ROC analysis of a large sample of septic patients at ED admission demonstrated that presepsin was superior to PCT and showed higher sensitivity, specificity, PPV, NPV and predictive accuracy in the early diagnosis of sepsis, which was in line with previous reports [4,5,9].

There was higher mortality as the severity of sepsis increased, and more than 50% mortality in severe sepsis [17]. Dellinger and colleagues, in the Early-Goal Directed Therapy in 2013 Guidelines of the Surviving Sepsis Campaign [27], recommended that a potential infection source should be confirmed as promptly as possible within the first 6 hours of presentation and that broad-spectrum antibiotic treatment must be administered within 1 hour after the recognition of severe sepsis and septic shock. Early identification of these high-risk patients is therefore crucial. In this study, ROC analysis showed that the AUC of presepsin (0.840) was markedly higher than that of PCT (0.741) (*P* <0.01) and displayed higher sensitivity (82.4%) and NPV (83.2%) in predicting severe sepsis. Meanwhile, in logistic regression analysis, presepsin was found to be an independent predictor of severe sepsis, but PCT was not, which further indicated that presepsin was superior to PCT in predicting severe sepsis.

Meanwhile, presepsin demonstrated high sensitivity (85.7%) and high NPV (96.3%) in predicting septic shock, and plasma presepsin levels lower than the above-mentioned cutoff value (550 pg/ml) may help rule out the possibility of septic shock. Additionally, in the logistic regression analysis, presepsin and PCT were found to be independent predictors of septic shock in septic patients, which indicated presepsin and PCT had certain value in predicting septic shock.

Scoring systems are also widely applied to risk stratification and prediction of prognosis in septic patients. In the present study, we found that MEDS score and APACHE II score increased along with progression of sepsis, reaching a maximum in septic shock. Similarly, MEDS score and APACHE II score were also a good parameter to reflect the severity of sepsis.

In this study, we further found that presepsin, PCT, MEDS score and APACHE II score were statistically significant for predicting severe sepsis, septic shock and 28-day mortality (all *P* <0.0001). The AUC of presepsin in combination with MEDS score or APACHE II score was significantly higher than MEDS score or APACHE II score alone in predicting severe sepsis (*P* <0.01), and although there was no difference compared with presepsin alone (all *P* >0.05), presepsin in combination with MEDS score or APACHE II score markedly increased AUC (0.875 or 0.858, respectively), which indicated that the combination of presepsin with MEDS score or APACHE II score obviously improved the accuracy of predicting severe sepsis.

Meanwhile, we also found the AUC of presepsin in combination with MEDS score or APACHE II score was significantly higher than presepsin alone in predicting septic shock (*P* <0.01). Although there was no difference compared with MEDS score or APACHE II score alone (all *P* >0.05), presepsin in combination with MEDS score or APACHE II score significantly increased the AUC (0.924 or 0.868, respectively). In addition, the AUC of presepsin in combination with MEDS score was significantly higher than that of presepsin in combination with APACHE II score (*P* <0.05). Taken together, combination presepsin with MEDS score or APACHE II score markedly enhanced the accuracy of predicting septic shock, and the combination of presepsin with MEDS score was superior to the combination of presepsin with APACHE II score in predicting septic shock.

Although the AUC of presepsin was slightly lower than that of PCT and MEDS score in predicting 28-day mortality in septic patients, no significant differences were found. However, the AUC of presepsin in combination with MEDS score or APACHE II score was significantly higher than presepsin alone (all *P* <0.01). Although the sensitivity (62.2%), the specificity (66.8%), the PPV (48.3%), and the NPV (78%) were a little low for presepsin for predicting 28-day mortality in septic patients, in the logistic regression analysis presepsin, MEDS score and APACHE II score were found to be independent predictors of 28-day mortality in septic patients, while PCT was not – this is in accordance with previous results from Ruiz-Alvarez and colleagues [28], suggesting presepsin was superior to PCT for predicting 28-day mortality in septic patients, and the combination of presepsin with MEDS score or APACHE II score significantly enhanced the accuracy of predicting 28-day mortality in septic patients.

Additionally, Spearman correlation analysis of presepsin found a significantly positive correlation with MEDS score and APACHE II score, indicating that presepsin, MEDS score and APACHE II score may facilitate evaluation of the severity of sepsis and allow effective risk stratification, as presepsin in combination with MEDS score or APACHE II score further enhanced the accuracy of evaluation of septic patients.

The median levels of presepsin were significantly higher in nonsurvivors than in survivors, which further confirmed that the higher the plasma presepsin level, the more adverse the outcome in septic patients. The plasma presepsin level was also a good index for evaluation of prognosis in septic patients.

### Limitations

Although the present study contained a relatively large sample size, some limitations merit consideration. First, it was a single-center study and did not compare with other biomarkers and severity score systems. Second, because it was difficult to obtain pathogen samples in an emergency room setting, blood culture and sputum sample were not taken and tested, and sepsis was established on the basis of clinical features, laboratory findings and imaging tests according to criteria for sepsis as defined by ACCP/SCCM.

## Conclusions

This study indicated that presepsin was a more valuable biomarker than PCT in the early diagnosis of sepsis, and presepsin in combination with MEDS score or APACHE II score significantly increased the prognostic accuracy in septic patients. Presepsin thus appears to be a promising biomarker and is superior to PCT for early diagnosis of sepsis, and risk stratification and evaluation of prognosis in septic patients in the ED.

## Key messages

● Plasma presepsin levels were a good parameter for reflecting the severity of sepsis and evaluating prognosis of septic patients.

● Presepsin was superior to PCT for diagnosing sepsis, and predicting severe sepsis, septic shock and 28-day mortality in septic patients in the ED.

● Presepsin, MEDS score and APACHE II score were all independent predictors of severe sepsis, septic shock and 28-day mortality in septic patients.

● Presepsin in combination MEDS score or APACHE II score markedly enhanced the predictive accuracy of predicting severe sepsis, septic shock and 28-day mortality in septic patients.

## Abbreviations

ACCP: American college of chest physicians; APACHE: Acute physiology and chronic health evaluation; AUC: Area under the receiver operating characteristic curve; ED: Emergency department; LR–: Negative likelihood ratio; LR+: Positive likelihood ratio; MEDS: Mortality in emergency department sepsis; NPV: Negative predictive value; PCT: Procalcitonin; PPV: Positive predictive value; ROC: Receiver operating characteristic; SCCM: Society of critical care medicine; SIRS: Systemic inflammatory response syndrome.

## Competing interests

The authors declare they have no competing interests.

## Authors’ contributions

C-SL conceived this study, designed the trial and obtained research funding. BL, Y-XC, QY and Y-ZZ conducted the trial and collected data. BL analyzed data, performed statistical analysis, drafted and revised the manuscript. C-SL takes responsibility for the paper as a whole. All authors read and approved the final manuscript.
